# Trastuzumab and paclitaxel in patients with EGFR mutated NSCLC that express HER2 after progression on EGFR TKI treatment

**DOI:** 10.1038/s41416-018-0194-7

**Published:** 2018-07-31

**Authors:** Adrianus J. de Langen, M. Jebbink, Sayed M. S. Hashemi, Justine L. Kuiper, J. de Bruin-Visser, Kim Monkhorst, Erik Thunnissen, Egbert F. Smit

**Affiliations:** 1 Department of Thoracic Oncology, Netherlands Cancer Institute-Antoni van Leeuwenhoek Hospital, Amsterdam, The Netherlands; 2 Department of Pulmonary Diseases, VU University Medical Center, Amsterdam, The Netherlands; 3 Department of Pathology, VU University Medical Center, Amsterdam, The Netherlands; 4 Department of Pathology, Netherlands Cancer Institute-Antoni van Leeuwenhoek Hospital, Amsterdam, The Netherlands

**Keywords:** Drug development, Targeted therapies, Non-small-cell lung cancer

## Abstract

**Background:**

HER2 expression and amplification are observed in ~15% of tumour biopsies from patients with a sensitising EGFR mutation who develop EGFR TKI resistance. It is unknown whether HER2 targeting in this setting can result in tumour responses.

**Methods:**

A single arm phase II study was performed to study the safety and efficacy of trastuzumab and paclitaxel treatment in patients with a sensitising EGFR mutation who show HER2 expression in a tumour biopsy (IHC ≥ 1) after progression on EGFR TKI treatment. Trastuzumab (first dose 4 mg/kg, thereafter 2 mg/kg) and paclitaxel (60 mg/m^2^) were dosed weekly until disease progression or unacceptable toxicity. The primary end-point was tumour response rate according to RECIST v1.1.

**Results:**

Twenty-four patients were enrolled. Nine patients were exon 21 L858R positive and fifteen exon 19 del positive. Median HER2 IHC was 2+ (range 1–3). For 21 patients, gene copy number by in situ hybridisation could be calculated: 5 copies/nucleus (*n* = 9), 5–10 copies (*n* = 8), and >10 copies (*n* = 4). An objective response was observed in 11/24 (46%) patients. Highest response rates were seen for patients with 3+ HER2 IHC (12 patients, ORR 67%) or HER2 copy number ≥10 (4 patients, ORR 100%). Median tumour change in size was 42% decrease (range −100% to +53%). Median duration of response was 5.6 (95% confidence interval [CI], 3.8 to 7.3) months. Treatment toxicity was mild with four patients experiencing grade ≥3 toxicity, including fatigue, neuropathy, neutropaenia, urinary tract infection, and pneumonitis.

**Conclusions:**

Trastuzumab-paclitaxel induces objective tumour responses in 46% of EGFR TKI pretreated patients with an activating EGFR mutation and HER2 expression. The treatment was well tolerated. The relation between response rate and HER2 expression level and copy number suggests effective HER2 targeting by trastuzumab, although the combination with paclitaxel does not allow to determine the relative contribution of the individual drugs in terms of treatment efficacy.

## Introduction

Non-squamous NSCLC that is characterised by an EGFR exon 19 deletion or an exon 21 L858R point mutation, is highly sensitive to treatment with EGFR tyrosine kinase inhibitors (TKIs). In metastatic patients, multiple studies have shown superior clinical outcome for treatment with first (erlotinib, gefitinib) or second (afatinib) generation EGFR TKIs, as compared to platinum doublet chemotherapy.^1^

However, almost all patients develop treatment resistance, typically 10–12 months after treatment initiation. Multiple resistance mechanisms have been described, involving three mechanisms: altered drug target, bypass track activation, and morphological alterations.^2^ The most frequent drug target alteration is the T790M mutation that accounts for 50–60% of secondary resistance to first and second generation EGFR TKI therapies.^2^ Based on phase III data, osimertinib (a third generation EGFR TKI) is the preferred treatment for patients whose tumour is T790M positive after progression on a first or second generation EGFR TKI.^3^

Another mechanism of treatment resistance is bypass track activation. This means that other transmembrane receptors or downstream kinases are altered, thereby bypassing inhibition of the EGFR growth signal. Tumor biopsies upon progression on EGFR TKI treatment have shown overexpression or amplification of HER2 and MET, and mutations in BRAF and PIK3CA.^2,4^ The third mechanism involves morphological alterations: dedifferentiation by epidermal-mesenchymal transition (EMT) or change to small-cell-lung carcinoma (SCLC), including RB1 loss.^2,4^

HER2 amplification and overexpression have been shown to be oncogenic in certain types of breast cancer and play an important role in the development and progression of disease. In order to activate downstream signalling, HER family members, including EGFR, HER2, HER3, and HER4 have to form dimers. The major partner for HER2 is EGFR and on this basis HER2 can modulate EGFR-signalling.^5^ Several reports suggest that HER2 signalling contributes to resistance to EGFR TKIs in EGFR-mutated patients. In vitro, it was demonstrated that acquired resistance to EGFR TKIs and monoclonal antibodies can be mediated by activation of HER2.^6,7^ This acquired resistance could be overcome by inhibition of HER2. Amplification of HER2 can be found in 12% of EGFR TKI-resistant cell lines^8^ and two clinical studies detected HER2-amplification in 13% of NSCLC-patients with acquired EGFR TKI resistance.^4,9^

Trastuzumab is a humanised IgG1 monoclonal antibody directed against HER2. It binds with high affinity and specificity to the extracellular domain of the HER2 protein, thereby suppressing HER2 signalling. As a result, it inhibits the growth of tumour cells and mediates antibody-dependent cellular cytotoxicity in cancer cells exhibiting HER2 protein expression.^10,11^ Trastuzumab is an approved treatment for breast-cancer and gastric-cancer patients and effective in the subgroup of patients with HER2 overexpression or amplification.^12,13^

When trastuzumab is combined with a taxane (e.g., paclitaxel or docetaxel), improved efficacy has been observed compared to trastuzumab monotherapy in breast cancer.^14,15^

Although multiple studies in NSCLC have failed to show benefit of HER2 directed treatment, these studies were performed in patients with HER2 expression, irrespective of the co-occurrence of an activating EGFR mutation and tested the hypothesis of HER2 being an oncogenic driver in NSCLC.^16–22^

We hypothesised that HER2 overexpression can drive therapeutic resistance in EGFR mutated NSCLC patients treated with EGFR TKIs and that these patients may benefit from treatment with trastuzumab-paclitaxel.

## Methods

A single centre single arm phase II study was designed to study the safety and efficacy of weekly trastuzumab-paclitaxel treatment in EGFR mutation positive NSCLC patients who showed tumour membrane HER2 expression in a tumour biopsy (IHC ≥ 1) after having experienced progressive disease on an EGFR TKI. The trial has been approved by the local ethics committee and registered on clinicaltrials.gov under the number NCT02226757.

### Eligibility criteria

Eligible patients had pathologically confirmed stage IV non-squamous NSCLC with an activating EGFR mutation and had progressed on EGFR TKI monotherapy. A biopsy obtained after progression on the last EGFR TKI had to be available and show HER2 expression (see below). Patients needed to have measurable disease according to RECIST v1.1 criteria, WHO performance status 0–2, willing and able to comply with the study prescriptions, be 18 years or older and able to give written informed consent. Key exclusion criteria were uncontrolled infectious disease, other active malignancy, major surgery in the previous 4 weeks, treatment with investigational drugs within the previous 4 weeks, known hypersensitivity to trastuzumab or paclitaxel, symptomatic brain metastases, a history of coronary artery disease or documented congestive heart failure, angina pectoris requiring medication, NYHA class III or IV, poorly controlled hypertension, clinically significant valvular heart disease, high risk for uncontrollable arrhythmias, or a left ventricular ejection fraction (LVEF) of <55%. Also patients needed to have adequate bone marrow, renal, and liver function.

### Pathology

For HER2 IHC, the primary antibody was rabbit monoclonal RTU clone 4B5 (Ventana, Roche) with antigen retrieval: 32 min CC1, incubation time 24 min. and Optiview DAB detection kit, according to the manufacturer. HER2 IHC was scored positive when distinct membranous staining was observed in at least 10% of the tumour cells and was categorised by staining intensity (1+ to 3+). In addition, the H score (0–300) was calculated.

For HER2 in situ hybridisation (ISH), the dual colour HER2/Ch17 DISH or the mono colour HER2 SISH kit from Ventana were used according to instruction from the manufacturer. The mean number of HER2 copies per nucleus was calculated after counting 40 tumour cell nuclei. The copy number variation (CNV) was distributed in three categories: 1–4, 5–10, and >10. The ratio with Ch17 was not used because the Ch17 copy number could not be calculated for the complete set of samples.

All biopsies were centrally reviewed prior to study inclusion by one of two experienced thoracic pathologists (E.T. or K.M.).

### Trial design and treatment

Patients were treated weekly with trastuzumab (first dose 4 mg/kg, thereafter 2 mg/kg) and paclitaxel (60 mg/m2) until disease progression, the development of unacceptable side effects, or a request by either the patient or the physician to discontinue treatment. All patients provided written informed consent before entering the trial.

Dose modifications were allowed for trastuzumab and paclitaxel. In case of cardiac dysfunction (LVEF decreases by ≥10 EF points and to <50%), trastuzumab was interrupted for 3 weeks. If a repeat LVEF measurement showed no improvement, trastuzumab was permanently discontinued. Administration of paclitaxel required neutrophil count to be ≥1.0 × 10^9^/L and platelet count ≥100 × 10^9^/L. In case of grade II peripheral neuropathy or any grade III toxicity paclitaxel was interrupted for 7 days (one cycle). In case of absence of improvement after 7 days or in case of grade IV neutropaenia or grade III mucositis at any time, the paclitaxel dose was decreased by 25%. Paclitaxel was discontinued in case of grade ≥3 peripheral neuropathy at any time.

### Trial end points

The primary end point was confirmed response rate according to RECIST v1.1, as determined by investigator assessment. Secondary objectives included toxicity according to CTC AE 4.0, disease control rate (DCR), median progression-free survival (PFS), duration of response (DOR) and overall survival (OS). Preplanned exploratory subgroup analyses for treatment outcome were performed for the different HER2 expression and gene copy number levels.

### Assessments

Baseline tumour assessments were performed within 28 days prior to initiation of study treatment. Brain imaging was required only in patients with known or suspected central nervous system (CNS) metastases. Baseline tumour measurements were done with CT of thorax, abdomen, and pelvis and followed by repeat CT scan every 6 weeks until progressive disease. In case of brain metastases, follow-up measurements included CT or MRI of the brain. Assessments for survival were performed every 6 weeks after objective disease progression or withdrawal from treatment. PFS was defined as the time from treatment initiation until the date of objective disease progression or death in the absence of progression. DOR was defined as the time from radiological response until the date of objective disease progression or death in the absence of progression. OS was defined as the time from study enrollment until death. Baseline assessments included lab tests (complete blood count including white blood cell differentiation, renal function, liver function, electrolytes, and thyroid function), pregnancy-test where implicated, clinical examination (blood pressure, heart rate, WHO performance status, weight, length), medical history, concomitant medication check, CTC AE 4.0, electrocardiogram (ECG), and multigated acquisition (MUGA) scan for assessing LVEF. Lab tests, clinical examination and CTC AE 4.0 were repeated every week. At week 12, 24, and 48, the MUGA scan was repeated.

### Statistical analysis

#### Sample size

It was postulated that it would be desirable to achieve a response rate of at least 30% since this would approach the response rate to platinum-based chemotherapy treatment,^23^ the alternative treatment option for most patients included in this trial. Thus the null hypothesis: H0 = 30% was tested versus the alternative of H1 > 30%. Eighty percent power would be achieved at a response of 53% with a one sided test of 10% (i.e., proportion under null hypothesis is 30% and under the alternative is 53%). The sample size required to achieve this is 20 patients. Estimation of the sample size was calculated by East (Version 6). Taken into account a lost to follow-up of 10%, it was suggested safe to include a total of 22 patients.

#### Primary study parameter

The study drug combination was deemed of interest for further drug development if at least 30% of patients would show an objective response (partial or complete response) on treatment. The number and proportions of responders and non-responders were calculated.

#### Secondary study parameters

Adverse events and serious adverse events were determined according to CTC AE 4.0.

PFS, DOR, and OS were analysed by the Kaplan–Meier method. Mean values with 95% CIs were calculated.

#### Exploratory study parameters

Subgroup analyses were based on the level of HER2 expression (1+ vs. 2+ vs. 3+) and the level of HER2 copy number (1–4 vs. 5–10 vs. >10).

## Results

From 08–2012 to 03–2017, 24 patients were enrolled. The inclusion of two additional patients than was anticipated was caused by enrollment of three patients at the same time, while there was a single slot left. After internal discussion with the ethical committee, it was allowed for all three patients to enter the study. Baseline patient demographics and disease characteristics are shown in Table 1. EGFR mutation type, including T790M, and HER2 expression level were known for all patients. HER2 copy number was known for 21 of 24 patients, because of limited tissue availability in three patients. Exon 19 deletion and exon 21 L858R point mutation were present in 63% and 37%, respectively. All patients received erlotinib or gefitinib as first EGFR TKI. A co-occurring exon 20 T790M mutation was present in 7/24 (29%) patients and all but one of these patients received a third generation EGFR TKI (rociletinib or osimertinib) on which they progressed prior to study enrollment. Most patients had 2+ or 3+ HER2 tumour membrane staining (range 1–3, median 2). HER2 copy number varied, with most tumours having more than five copies per cell nucleus. The median time from diagnosis of stage IV non-small-cell lung cancer to study enrollment was 16.5 months (range 6 months–5.6 years). Twenty-five percent of patients was treated with platinum doublet chemotherapy and 75% received two or more lines of treatment prior to enrollment. With the exception of one patient, all patients received treatment with an EGFR TKI at the moment of study enrollment. The single outlier received pemetrexed monotherapy for 3 months after progressing on osimertinib treatment and had no response to pemetrexed therapy.

**Table 1:** Baseline patient characteristics

Patient characteristics	All patients (*n* = 24)
Median age (range)–year	68 (40–82)
Performance score	
0	4
1	13
2	7
Female sex–no. (%)	17 (71)
Ethnicity–no. (%)	
Caucasian	19 (79)
Asian	5 (21)
Type of EGFR mutation(s)–no. (%)	
Exon 19 del	10 (42)
Exon 21 L858R	7 (29)
Exon 21 L858R + exon 20 T790M	2 (8)
Exon 19 del + exon 20 T790M	5 (21)
Brain metastases at baseline–no. (%)	
Yes and treated with radiotherapy	4 (17)
Yes and untreated	4 (17)
No	16 (66)
No. of previous anticancer regimens for advanced disease (number and range)	2 (1–8)
1	6
2	11
≥3	7
Last line of EGFR-TKI–no. (%)	
Gefitinib	4 (17)
Erlotinib	8 (33)
Afatinib	0 (0)
Osimertinib	9 (38)
Rociletinib	3 (12)
HER2 IHC–no. (%)	
1	3 (12)
2	9 (38)
3	12 (50)
HER2 GCN–no.	
<5	9 (43)
5–10	8 (38)
≥10	4 (19)

### Efficacy

**Fig. 1:**
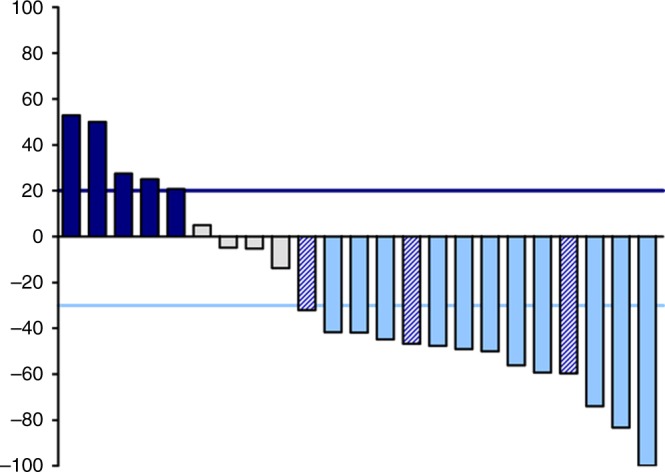
Waterfall plot showing maximum percentage change from baseline in tumour size. Grey bars represent patients with a partial or complete response, white bars represent patients with stable disease as best response and black bars represent patients with progressive disease. Grey bars with stripes represent patients with progressive disease due to isolated progression in the brain and >30% decrease in size of the extracerebral lesions. All responses were assessed with RECIST v1.1

Of the 24 patients treated with trastuzumab and paclitaxel, 11 (46%) had a confirmed objective response with one complete response (Table 2). Median tumour change in size was 42% decrease (range −100% to +53%; Fig. 1). Of the objective responses, 64% were observed after six weeks of treatment and 100% after 12 weeks. At the time of data cutoff, the median follow-up for all patients was 2.3 years. The median duration of confirmed objective response was 5.6 (95% CI 3.8–7.3) months with all patients having experienced disease progression. Median time on treatment (until progression) was 7.0 (95% CI 4.6–9.3) months for patients with a confirmed objective response and 2.3 (95% CI 0–5.8) months for all patients.

**Table 2:** Drug-related adverse events occurring in >20% of patients and all grade ≥3 drug-related events grouped by preferred term according to CTC AE 4.0

Adverse event	Grade 1 *n* (%)	Grade 2 *n* (%)	Grade 3 *n* (%)
Fatigue	9 (38)	0 (0)	1 (4)
Myalgia	9 (42)	0 (0)	0 (0)
Dyspnea	5 (21)	2 (8)	0 (0)
Neuropathy	4 (21)	1 (4)	1 (4)
Headache	6 (25)	0 (0)	0 (0)
Constipation	5 (21)	0 (0)	0 (0)
Nausea	5 (21)	0 (0)	0 (0)
Neutrophil count decreased	3 (13)	0 (0)	1 (4)
Pneumonitis	0 (0)	0 (0)	1 (4)
Urinary tract infection	1 (4)	0 (0)	1 (4)

Three of nine patients with osimertinib as last treatment (33%) progressed due to isolated progression in the brain, while the extracerebral lesions decreased in size by >30% (Fig. 1).

**Fig. 2:**
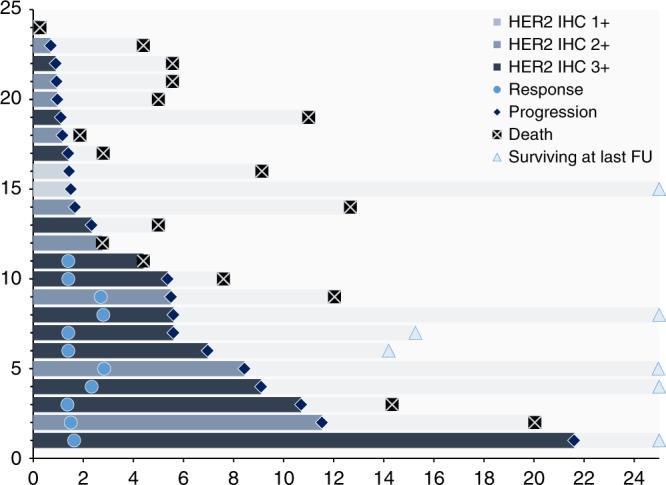
Swimmer plot representing the survival, progression-free survival, and response kinetics. Time on *x*-axis is time in months. On the *y*-axis every bar represents a single patient

Four patients (17%) had SD as best response after six weeks, of which two showed progression on the subsequent scan after 12 weeks of treatment. Nine patients (38%) suffered from disease progression (*n* = 8) or were not evaluable (*n* = 1) for response evaluation. Overall median PFS was 2.3 (95% CI, 0–5.8) months, while this was 1.1 (95% CI, 0.7–1.5) months and 5.4 (95% CI, 1.4–9.4) months for patients with osimertinib or other TKI as last treatment, respectively. Individual outcome parameters are presented in Fig. 2.

### Tolerability and adverse event profile

Treatment-related adverse events were observed in 62% of patients, with the most common being fatigue (42%), dyspnea (29%), neuropathy (29%), constipation (21%), and nausea (21%). Grade 4 and 5 events were not observed. Grade 3 drug-related events were encountered in 4 (17%) patients, with one patient having experienced two grade 3 events (Table 2). Dose interruption for paclitaxel (≥1 cycle) was necessary in 4 (17%) patients. Overall, one patient discontinued therapy (both drugs) due to grade 3 pneumonitis, one patient discontinued paclitaxel due to grade III neuropathy and in one patient the paclitaxel dose was reduced to 75% because of persisting grade II neuropathy. LVEF was ≥55% for all patients at baseline and in none of the patients a decrease of ≥15% was observed during follow-up.

### Relation between HER2 expression and gene copy number and patient outcome

HER2 expression level was known for all patients, while gene copy number could be calculated for 21 patients. There was no relation between the level of HER2 expression and HER2 copy number (*p* = 0.37). HER2 expression level and gene copy number were both related to patient outcome in terms of response rate and progression-free survival. The highest response rates were observed in patients (*n* = 4) with more than 10 gene copies per nucleus (ORR 100%) and in patients (*n* = 12) with 3+ HER2 expression (ORR 67%). None of the patients with 1+ HER2 expression responded, while this was 22% for the subgroup with a gene copy number of less than 5 (Table 3 and Table S1).

**Table 3:** Response to treatment (intention-to-treat population)

Response	Overall population (*n* = 24)	HER2 IHC 1 (*n* = 3)	HER2 IHC 2 (*n* = 9)	HER2 IHC 3 (*n* = 12)	HER2 GCN < 5 (*n* = 9)		HER2 GCN 5–10 (*n* = 8)		HER2 GCN ≥ 10 (*n* = 4)	
Type of response										
Complete	1	0	0	1		0		0		1
Partial	10	0	3	7		2		4		3
Stable disease ≥6 weeks	4	1	2	1		3		0		0
Progression	8	1	4	3		3		4		0
Death	1	1	0	0		1		0		0
Objective response rate (95% CI)	46% (26–67%)	0% (0–71%)	33% (7–70%)	(35–90%)		(3–60%)		50% (16–84%)		100% (40–100%)
Disease control rate after 6 weeks (95% CI)	63% (41–81%)	33% (1–91%)	56% (21–86%)	75% (43–95%)		56% (21–86%)		50% (16–84%)		100% (40–100%)
Duration of response										
Median no. of months	5.6	n.a.	5.6	4.2		2.8		3.0		4.2
(95% CI)	(3.8–7.3)		(1.1–10.1)	(2.0–6.4)		n.a.		(0.3–5.7)		(1.5–6.9)
Progression-free survival										
Median no. of months	2.3	1.4	1.7	5.4		1.7		1.4		5.6
95% CI	(0–5.8)	(0–3.3)	(0.2–3.1)	(4.0–6.7)		(0–3.3)		(0–5.6)		(1.9–9.3)

*GCN* gene copy number

A trend towards longer PFS was seen for patients with HER2 IHC 3+ vs. HER2 IHC ≤2+ (5.4 [95% CI 4.0–6.7] months vs. 1.4 [95% CI 0.9–2.0] months; *p* = 0.15), and patients with a GCN ≥ 10 vs. GCN < 10 (5.6 [95% CI 1.9–9.3] months vs. 1.7 [95% CI 0.4–2.9] months; *p* = 0.05).

### HER2 expression and amplification dynamics over time during EGFR TKI treatment

In order to investigate whether the observed HER2 overexpression or amplification after progression on EGFR TKI treatment was induced by EGFR TKI treatment or if this was already present in the tumour sample prior to EGFR TKI treatment, the pre-EGFR TKI biopsy samples, where available, were analysed for HER2 expression with IHC.

For 17/24 (71%) patients it was possible to analyse the diagnostic tumour sample prior to any treatment. For these 17 patients with paired biopsies, the median HER2 IHC and H scores prior to any treatment were 2+ and 100, while these were 3+ and 240 for the post EGFR TKI tumour samples, respectively, suggesting that EGFR TKI treatment either induced or increased HER2 expression (Fig. S1).

### Post-treatment resistance profiling and follow-up treatment

Of the 11 patients with an objective partial or complete response, ten showed progression on follow-up radiological assessment and one died while still in remission based on recent radiological assessment. Of these ten patients, six underwent a rebiopsy upon progression. One patient had isolated leptomeningeal disease progression and could not be rebiopsied. In four out of six biopsies HER2 was negative by IHC.

Most patients (71%) received subsequent therapy after disease progression and one patient received study treatment beyond progression because of isolated brain progression with an extracerebral partial response. Isolated brain progression with extracerebral stable disease (*n* = 2) or >30% size decrease of target lesions (*n* = 3) occurred in 21% of patients, suggestive of limited activity of trastuzumab-paclitaxel in the brain. Median overall survival in the intention-to-treat population from the time of study drug initiation to death was 7.0 (95% CI 0–14.2) months, with 7 patients being censored.

## Discussion

This trial demonstrates that trastuzumab and paclitaxel treatment can induce objective tumour responses in patients with EGFR mutation positive NSCLC and HER2 expression after progression on EGFR TKI treatment. This is the first trial that evaluates HER2 targeting in this group of patients. The confirmed objective response rate of 46%, with a median duration of response of 5.6 months. Although this is beyond what can be expected with single agent taxane monotherapy in this patient population.^24^ The relative contribution of trastuzumab in terms of treatment efficacy cannot be determined due to the trial design with trastuzumab and paclitaxel combination therapy. Although trastuzumab monotherapy would have been a purer trial design, patients enrolled in this study were eligible for chemotherapy outside a clinical trial and withholding patients for any form of chemotherapy was regarded unethical at the time of trial design. Also, the addition of paclitaxel to trastuzumab was found to be superior to trastuzumab monotherapy in breast cancer.^25–27^

Although, the relatively low number of patients included in this trial (*n* = 24) does not allow for formal subgroup analyses, exploratory subgroup analyses for HER2 IHC and ISH were predefined in the study protocol. A gradual increase in response rate and PFS was observed with increasing HER2 expression and copy number. HER2 expression was absent in four of six post-progression tumour samples of responding patients. These findings suggest effective HER2 targeting and supports preclinical findings of HER2 as a targetable driver of EGFR TKI resistance.

Because HER2 overexpression (IHC 2+ and 3+) is observed in 15–30% of lung adenocarcinoma^28–31^ and HER2 3+ and/or amplification is observed in 2–6% of lung adenocarcinoma,^28–32^ the pre-EGFR TKI treatment tumour samples were analysed for HER2 expression as well. In 17 patients with the paired biopsies we found that 8/17 (47%) patients were already IHC 3+ prior to any treatment, while 8/17 (47%) patients were either negative for HER2 IHC or showed an increase in the HER2 IHC score (Fig. S1A). Interestingly, looking at the HER2 H score, almost all patients showed an increase in the H score after EGFR TKI treatment (Fig. S1B), suggestive of treatment induced selection of an HER2 driven resistant cell population. Emerging data supports the scenario whereby the selective pressure imposed by the chronic exposure to EGFR TKIs leads to the expansion of pre-existing resistant clones carrying specific genetic alterations, which may ultimately become dominant.^33^

Despite the high response rate and relatively long duration of response, median progression-free survival (2.3 months) was short and worse than the PFS being observed with platinum-doublet chemotherapy after progression on EGFR TKI treatment in the IMPRESS study (5.4 months).^23^ Of the patients that received chemotherapy after progression on EGFR TKI treatment in the current study (*n* = 7), the median PFS during chemotherapy treatment was 4.0 (95% CI 0–10.5) months. The short PFS during trastuzumab-paclitaxel observed in this trial was mainly due to a substantial number of patients with progressive disease as best response (*n* = 9). An interesting and important observation was the heterogeneous response in three patients with progression in the brain and >30% size decrease of the target lesions outside the CNS. All three patients received osimertinib as last treatment before study enrollment. A brain MRI was not part of the baseline screening assessments. This observation suggests that the combination of trastuzumab and paclitaxel is less active in controlling brain metastases, especially in patients with osimertinib as last treatment, an EGFR TKI with excellent CNS penetration and efficacy.^3,34^ The cause for this might be a sudden loss of tumour control in the brain, caused by osimertinib discontinuation and the limited CNS bioavailability of trastuzumab and paclitaxel due to the blood–brain barrier. Another explanation could be a different resistance mechanism in brain metastases compared to that of the extracerebral tumour lesions.

Looking at the extracerebral objective response rate, this study shows that trastuzumab and paclitaxel combination therapy is active in controlling HER2 expression and EGFR mutation positive NSCLC outside the CNS with an extracerebral response rate of 58%.

Heterogeneous responses were observed in two patients with a decrease of multiple lesions and progression of other lesions. Upon reintroduction of the EGFR TKI (osimertinib), both patients experienced a decrease of the progressing lesions, confirming the presence of a disease flair after EGFR TKI discontinuation that could not be suppressed by trastuzumab-paclitaxel. This observation suggests that a subset of patients with HER2 bypass track activation may benefit from co-inhibition of EGFR, especially patients that progress on osimertinib.

The safety profile of weekly trastuzumab-paclitaxel was consistent with that observed in the breast cancer setting^35^ and holds favourably when compared with 3-weekly taxane monotherapy^36^ or platinum-doublet chemotherapy,^37,38^ the alternative therapeutic options for this population.

Although the response rate and duration of response are encouraging, an important subset of patients did not fare well on the treatment. In addition, due to the design of this study with co-treatment with trastuzumab and paclitaxel it remains an open question on what the relative contribution of both drugs has been in terms of treatment efficacy.

In subsequent studies targeting HER2 bypass resistance, we suggest to co-target HER2 and EGFR by continuing EGFR TKI treatment, especially in case of osimertinib because of its efficacy in controlling CNS metastases. The use of trastuzumab-emtansine (T-DM1) as HER2 targeting drug would allow to selectively target HER2 without a systemic cytotoxic drug, while retaining the synergistic antitumour effects of trastuzumab and an anti-microtubulin cytotoxic drug. In breast cancer, T-DM1 efficacy was equivocal to trastuzumab-paclitaxel treatment with less toxicity^8^ and therefore T-DM1 can be used as a taxane-free treatment regimen. In EGFR mutation positive NSCLC, in-vitro experiments suggest that T-DM1 can overcome HER2 bypass track resistance in patients with EGFR mutated NSCLC.^39^ Two clinical studies evaluated T-DM1 treatment in NSCLC patients with HER2 alterations.^40,41^ However, both studies enrolled patients with HER2 as driver of tumourigenesis, and not necessarily as driver of TKI resistance. Stinchcombe et al. evaluated T-DM1 in patients with HER2 overexpression.^40^ No responses were seen in patients with HER2 2+ tumour expression (*n* = 29), while an objective response rate of 20% was seen for patients with HER2 3+ tumour expression (*n* = 20). Two patients with an EGFR mutation were included and one of these patients showed a partial response. Hotto et al. evaluated T-DM1 in patients with HER2 overexpression (*n* = 8) or mutation (*n* = 7).^41^ The objective response rate in this study was 7%. Two EGFR mutation positive patients were included and these patients did not show a response. These studies show that T-DM1 has limited efficacy in HER2 expression, amplification and/or mutation positive NSCLC. The number of patients with a co-occuring EGFR mutation in these studies seems to be too low to draw conclusions.

In conclusion, the current study shows that trastuzumab-paclitaxel induces objective tumour responses in an important number of patients with EGFR mutation positive NSCLC that show HER2 expression after progression on an EGFR TKI. HER2 expression level and gene copy number both seem to be related to the level of benefit. Future studies are needed to evaluate the relative contribution of trastuzumab in terms of antitumour efficacy in this disease setting.

## Electronic supplementary material

Table S1

Figure S1A

Figure S1B

Color artwork production form

## **Availability of data and material:**

The datasets generated during and/or analyzed during the current study are on file with the corresponding author and information can be requested by contacting the corresponding author.
