# Supplementary material for: Trastuzumab and paclitaxel in patients with EGFR mutated NSCLC that express HER2 after progression on EGFR TKI treatment
Source: Br J Cancer. 2018 Jul 31;119(5):558–64. doi: 10.1038/s41416-018-0194-7 (PMC6162232; doi:10.1038/s41416-018-0194-7)
Supplement: Supplementary file 1 — Table S1 [file 41416_2018_194_MOESM1_ESM.docx]

| **Patient number** | **Gender** | **Age** | **EGFR mutation** | **Platinum-doublet Cx** | **Last EGFR TKI** | **HER2 IHC** | **HER2 ISH** | **Best response** | **PFS (months)** |
| --- | --- | --- | --- | --- | --- | --- | --- | --- | --- |
| 1 | F | 40 | Ex 19 del | No | gefitinib | 3 | >10 | CR | 9.1 |
| 2 | M | 71 | Ex 19 + T790M | No | osimertinib | 3 | 3 | PR | 7.0 |
| 3 | M | 82 | Ex 21 + T790M | No | osimertinib | 3 | 5 | PR | 4.4 |
| 4 | F | 72 | Ex 21 | No | osimertinib | 3 | >10 | PR | 5.6 |
| 5 | M | 64 | Ex 21 | Yes | erlotinib | 3 | >10 | PR | 5.4 |
| 6 | F | 66 | Ex 19 + T790M | Yes | gefitinib | 2 |  | PR | 11.5 |
| 7 | M | 58 | Ex 19 | No | erlotinib | 3 | 4 | PR | 5.6 |
| 8 | F | 67 | Ex 19 | No | rociletinib | 2 | 5 | PR | 5.5 |
| 9 | F | 73 | Ex 21 + T790M | No | rociletinib | 2 | 5 | PR | 8.4 |
| 10 | M | 54 | Ex 19 | No | erlotinib | 3 | 6 | PR | 10.7 |
| 11 | F | 70 | Ex 21 | No | gefitinib | 3 | >10 | PR | 21.6 |
| 12 | F | 45 | Ex 19 + T790M | Yes | rociletinib | 1 |  | SD | 1.5 |
| 13 | F | 81 | Ex 21 | No | erlotinib | 2 | 3 | SD | 1.7 |
| 14 | M | 75 | Ex 19 | No | erlotinib | 3 | 3 | SD | 2.3 |
| 15 | F | 76 | Ex 19 | No | erlotinib | 2 | 3 | SD | 2.8 |
| 16 | M | 79 | Ex 19 + T790M | Yes | osimertinib | 2 | 5 | PD | 0.7 |
| 17 | M | 74 | Ex 19 + T790M | Yes | gefitinib | 2 |  | PD | 1.2 |
| 18 | F | 64 | Ex 21 | Yes | osimertinib | 3 | 2 | PD | 0.9 |
| 19 | F | 72 | Ex 21 | No | erlotinib | 1 | 8 | PD | 1.4 |
| 20 | F | 69 | Ex 19 | No | osimertinib | 3 | 8 | PD | 1.4 |
| 21 | F | 62 | Ex 21 | No | osimertinib | 2 | 3 | PD* | 0.9 |
| 22 | F | 66 | Ex 19 | No | osimertinib | 3 | 4 | PD* | 1.1 |
| 23 | F | 54 | Ex 19 | No | osimertinib | 2 | 7 | PD* | 1.0 |
| 24 | F | 53 | Ex 19 | No | erlotinib | 1 | 3 | n.a. | 0.3 |
