# Supplementary figures and images for: Trastuzumab and paclitaxel in patients with EGFR mutated NSCLC that express HER2 after progression on EGFR TKI treatment
Source: Br J Cancer. 2018 Jul 31;119(5):558–64. doi: 10.1038/s41416-018-0194-7 (PMC6162232; doi:10.1038/s41416-018-0194-7)

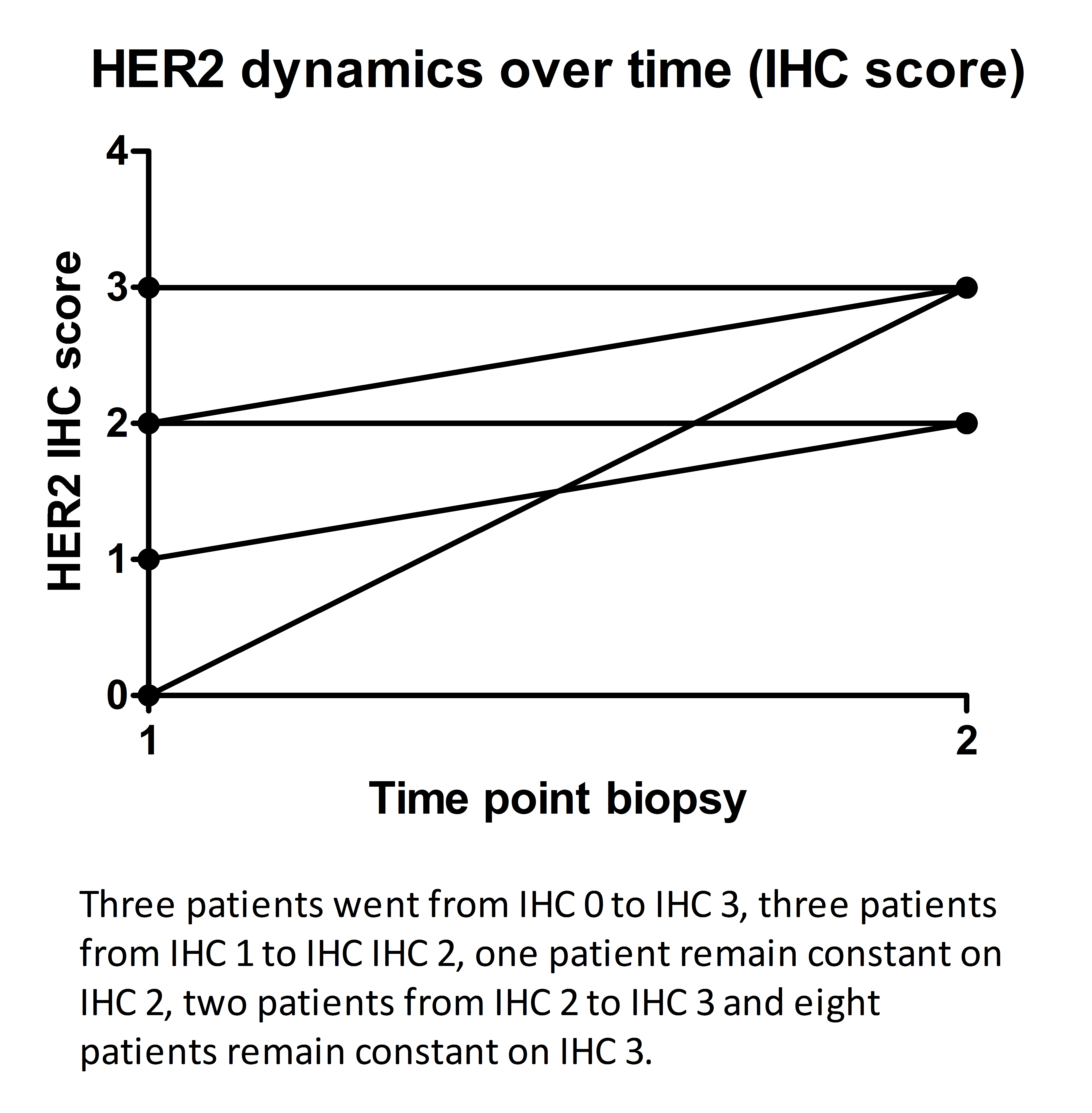

Supplement: Supplementary file 2 — Figure S1A [file 41416_2018_194_MOESM2_ESM.jpg]

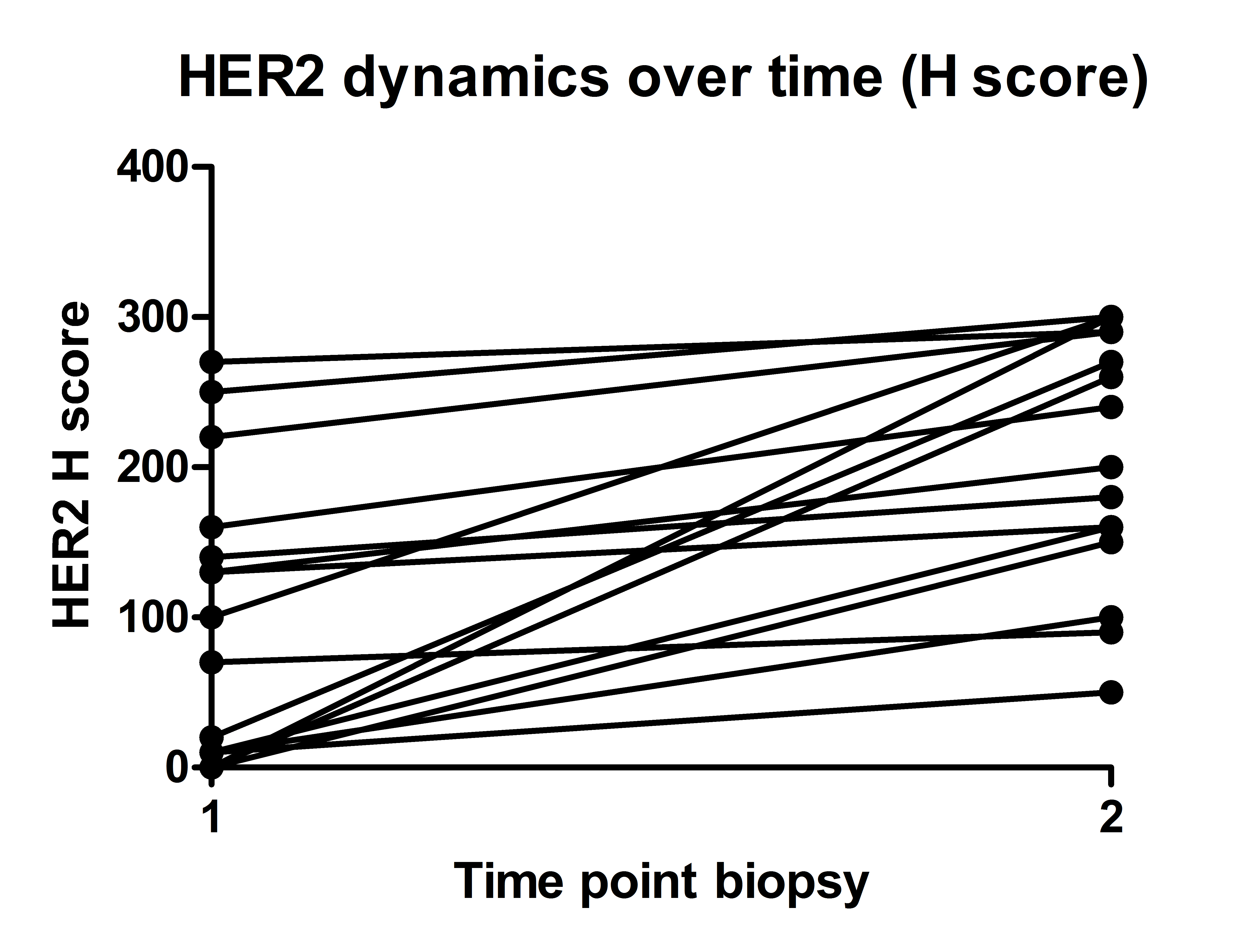

Supplement: Supplementary file 3 — Figure S1B [file 41416_2018_194_MOESM3_ESM.jpg]
