# Supplementary material for: Trastuzumab and paclitaxel in patients with EGFR mutated NSCLC that express HER2 after progression on EGFR TKI treatment
Source: Br J Cancer. 2018 Jul 31;119(5):558–64. doi: 10.1038/s41416-018-0194-7 (PMC6162232; doi:10.1038/s41416-018-0194-7)
Supplement: Supplementary file 4 — Color artwork production form [file 41416_2018_194_MOESM4_ESM.docx]

**Table S1. Individual patient and tissue characteristics, including HER2 expression level and copy number, and outcome to study treatment**

* Patients with progression in the brain in the presence of a decrease in size by >30% of the extracerebral lesions.

# Censored patients (alive at time of data cut-off; May-23-2018).

Platinum-doublet Cx: whether patients received platinum-doublet chemotherapy for their disease anywhere prior to study enrollment.

**Figure S1. HER2 expression dynamics over time. HER2 IHC (Figure S1A) and HER2 H (Figure S1B) scores were assessed for the tumor biopsies prior to any treatment (time point 1) and after EGFR TKI treatment (time point 2).**
